# Exploring gut microbiota and spinal cord injury: pathogenesis, treatment strategies and prospects

**DOI:** 10.3389/fimmu.2025.1693883

**Published:** 2025-12-15

**Authors:** Qin Chen, Mingliang Zhong, Ye Lin

**Affiliations:** 1 Department of Spine Surgery, Ganzhou Hospital-Nanfang Hospital, Southern Medical University (Ganzhou People’s Hospital), Ganzhou, Jiangxi, China; 2 Department of Infectious Disease, Ganzhou Hospital-Nanfang Hospital, Southern Medical University (Ganzhou People’s Hospital), Ganzhou, Jiangxi, China

**Keywords:** spinal cord injury, gut microbiota dysbiosis, probiotics, treatment strategies, fecal microbiota transplantation

## Abstract

Spinal cord injury (SCI) is a disabling central nerve system (CNS) injury, often caused by factors such as traffic accidents, falls from heights, violent trauma, and sports injuries, commonly resulting in permanent loss of motor and sensory function below the level of injury. Increasing evidence suggests that gut microbiota influences the occurrence and development of CNS diseases through the brain-gut axis. Recent studies indicate that patients with SCI frequently exhibit gut microbiota dysbiosis. Changes in gut microbiota can lead to gut barrier disruption, triggering neurogenic inflammatory responses, thereby hindering recovery after SCI, while reshaping gut microbiota may benefit the recovery of intestinal function and neurofunction after SCI. In this review, we summarize emerging literature on the role of microbiota after SCI. We elucidate the intrinsic connection between gut microbiota and SCI, explore the role of gut microbiota in the pathogenesis of SCI, and investigate potential intervention strategies targeting gut microbiota, including probiotic therapy, fecal microbiota transplantation (FMT), and regulation of metabolites, aiming to provide theoretical basis and translational prospects for developing innovative microecological targeted therapeutic approaches.

## 1 Introduction

SCI is a highly destructive CNS injury disease, often triggered by factors such as traffic accidents, falls from heights, violent trauma, and sports injuries (1, 2). It has been reported that the annual incidence of SCI worldwide is estimated at 250,000 to 500,000 cases, with a disability rate as high as 70% (3). Due to the very weak autonomous repair ability of nerves after SCI, the damaged spinal cord has difficulty in tissue repair and functional reconstruction (4, 5). This not only places a significant burden on patients but also poses great challenges to healthcare workers (6). The pathological process of SCI is usually divided into two stages: primary injury and secondary injury (7). Primary injury refers to the immediate destruction of the structure and function of the spinal cord caused by external forces acting directly or indirectly on it (8). Secondary injury, on the other hand, refers to a series of subsequent injuries triggered secondary to primary injury, including spinal cord edema, hemorrhage, inflammatory response, oxidative stress, neuronal and glial cell death, axonal demyelination, and glial scar formation, among other complex and interrelated pathological reactions (9–11). These pathological changes collectively make the neural repair process after SCI face numerous difficulties. Currently, the treatment methods for SCI mainly include early surgical decompression, symptomatic supportive treatment with neuroprotective drugs, and rehabilitation exercises (12). However, due to the extremely complex pathological process of SCI and the microenvironment of neural repair, these interventions remain insufficient to achieve a complete cure and full restoration of neural function (13).

**Figure 1:**
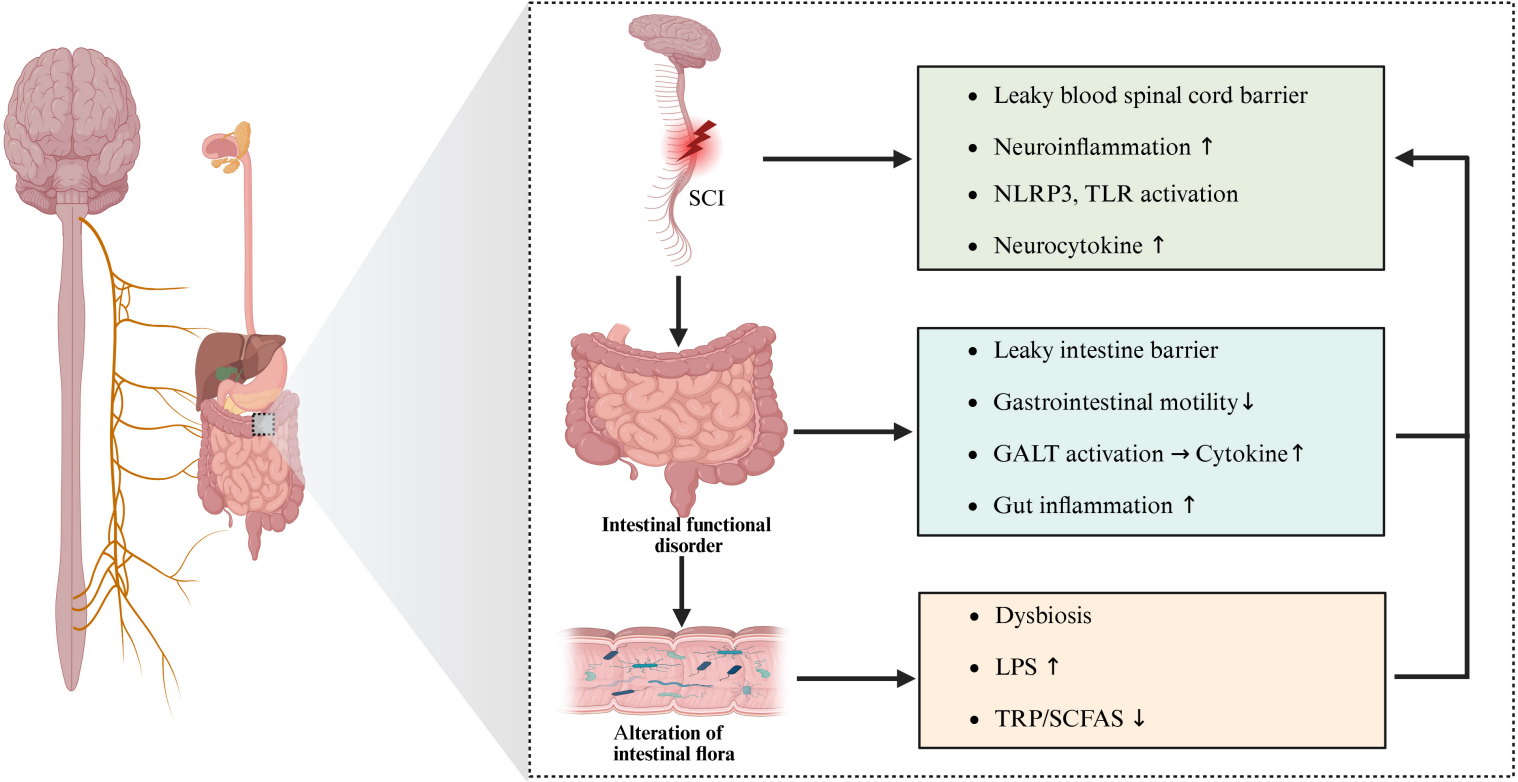
Bidirectional regulation of SCI and gut microbiota. SCI leads to neuroinflammation, including excessive release of NLRP3 inflammasome, TLRs, and inflammatory factors, as well as damage to the spinal cord barrier. In addition, SCI causes autonomic dysfunction, which leads to altered intestinal motility and intestinal immune dysfunction. These changes result in dysbiosis of the gut microbiota and damage to the gut barrier. An imbalance of gut microbiota can exacerbate intestinal inflammation. Leakage of gut microbial components and their metabolic product, LPS, may enter the CNS through peripheral blood circulation and damaged intestinal and blood-brain barriers. This translocation aggravates neuroinflammation and hinders functional recovery from SCI. (SCI, Spinal cord injury; CNS, Central nerve system; NLRP3, NLR family pyrin domain-containing-3; TLRs, Toll-like receptors; TRP, Tryptophan; SCFAs, Short-chain fatty acids; GALT, Gut-Associated lymphoid tissue; LPS, Lipopolysaccharide).

In recent years, with the proposal and continuous refinement of the “gut microbiota-gut-brain” axis theory, the role of gut microbiota in CNS diseases has gradually become a research hotspot (14). The gut microbiota, a large and complex microbial community in the human intestine, forms a mutually beneficial symbiotic relationship with the host. It plays a crucial role in physiological functions such as digestion, nutrient absorption, and immune regulation. Increasing evidence suggests a close connection between gut microbiota and the CNS, with bidirectional interactions occurring through neural, immune, and endocrine pathways, collectively known as the “gut microbiota–gut–brain” axis (15, 16). The spinal cord, as an important component of the CNS, has also attracted attention regarding its potential association with gut microbiota in SCI. Studies have found that SCI patients often exhibit bowel dysfunction and gut microbiota dysbiosis (17). This imbalance may negatively affect the recovery of neurological function after SCI through multiple mechanisms. These include exacerbating the destruction of the intestinal barrier, which allows harmful substances from the intestine to enter the bloodstream and trigger systemic inflammatory responses. Consequently, neuroinflammation is aggravated, hindering neurological repair and the recovery from SCI-related complications, thereby impairing neural regeneration and axonal remodeling (18, 19) (**Figure 1**). Therefore, in-depth research on the interaction mechanisms between gut microbiota and SCI is of great theoretical and practical significance for elucidating the pathogenesis of SCI, identifying new therapeutic targets, and developing more effective treatment strategies.

This review systematically outlines the pathogenesis of SCI, clarifies the association between gut microbiota and SCI and its role in disease progression, and focuses on the intrinsic connection of the “gut microbiota–spinal cord” axis by analyzing the mechanistic impact of microbiota dysregulation on SCI progression. On this basis, we propose microbiota-targeted intervention strategies centered on microbiome modulation, aiming to provide a mechanistic framework and evidence to support the clinical translation of SCI therapies.

## 2 SCI and gut microbiota dysbiosis

### 2.1 Gut microbiota

The gut microbiota refers to the vast and diverse community of microorganisms residing in the human gut, which forms a mutually beneficial symbiotic relationship with the host and plays a crucial role in human health. The number of microorganisms in the gut of adults can reach 10^14^, about ten times the number of human somatic cells. The number of genes they contain is approximately 100 times that of the human body itself, and the gut microbiota is thus commonly referred to as the “second genome” of the human body (20–23).

From a classification perspective, the gut microbiota can be classified into dozens of phyla, including Firmicutes, Bacteroidetes, Proteobacteria, Actinobacteria, and others (24). Notably, Firmicutes and Bacteroidetes are the dominant phyla in the gut microbiota and play a key role in maintaining gut microbial homeostasis (25). For example, many bacteria in the Firmicutes phylum can produce short-chain fatty acids (SCFAs), providing energy for intestinal epithelial cells and promoting normal development and functional maintenance of the gut (26). Bacteroidetes, on the other hand, are involved in the digestion of food and the absorption of nutrients, significantly impacting the host’s metabolic health (27). Based on their relationship with the host, the gut microbiota can be divided into symbiotic bacteria, opportunistic pathogens, and pathogens. Symbiotic bacteria are beneficial microorganisms that have coevolved with the host over a long evolutionary timescale. They aid the host in digesting complex carbohydrates, synthesizing vitamins, and enhancing immunity (28). Bifidobacterium and Lactobacillus are typical representatives of symbiotic bacteria. Bifidobacterium can ferment carbohydrates to produce lactic acid and acetic acid, lowering the intestinal pH and inhibiting the growth of harmful bacteria (29). Lactobacillus can produce various antimicrobial substances, maintaining the microbial balance of the gut (30). Opportunistic pathogens are harmless to the host under normal circumstances, but under specific conditions, such as decreased host immunity or disruption of gut microbial balance, they may overgrow and cause diseases (31). Escherichia coli, when present in normal quantities in the gut, aids in digestion and nutrient absorption; however, when the gut microbial balance is disrupted, it may translocate to other tissues and organs, leading to infections (32). Pathogens are bacteria pathogenic to the host; once they proliferate excessively in the gut, they can disrupt normal gut function and cause various diseases (33). For example, Salmonella can cause food poisoning, leading to symptoms such as diarrhea, abdominal pain, and vomiting (34). Based on their oxygen requirements, the gut microbiota can be divided into strict anaerobes, facultative anaerobes, and aerobes. Strict anaerobes, such as Clostridium species, are the main constituents of the gut microbiota and thrive in an anaerobic environment. These bacteria are involved in many important metabolic processes, such as the break down of cellulose and the production of SCFAs (35, 36). Facultative anaerobes can survive in both aerobic and anaerobic conditions. Escherichia coli belongs to this category, as it can utilize oxygen for aerobic respiration in the gut and also perform anaerobic fermentation under hypoxic conditions (37). Aerobes are relatively few in number in the gut, but they also play a role in gut microbial ecology. Some aerobes consume oxygen in the gut, creating a suitable environment for strict anaerobes (38).

The gut microbiota plays an indispensable role in the digestion and nutrient absorption processes of the human body. It helps break down indigestible components in food, such as dietary fiber, converting them into substances like SCFAs that can be absorbed and utilized by the body (39). SCFAs provide energy to intestinal epithelial cells, promote the growth and repair of the intestinal mucosa, and regulate the immune function of the gut, thereby maintaining intestinal health (40). The gut microbiota also participates in the synthesis of vitamins, particularly vitamin K (such as vitamin K2, menaquinone), and B vitamins (such as folate B9, biotin B7, and cobalamin B12) (41). These vitamins are crucial for normal physiological processes in the body, including coagulation, energy metabolism, and nervous system function (42). Among them, vitamin K2 is an important fat-soluble vitamin produced by gut microbiota (such as Bacteroides and Lactobacillus). It is primarily involved in the γ-carboxylation reaction and serves as an essential coenzyme for the normal synthesis of coagulation factors II, VII, IX, and X (43, 44). Additionally, vitamin K2 regulates bone metabolism by promoting calcium deposition through the activation of osteocalcin, thereby maintaining bone health. Furthermore, it exerts protective effects in the nervous and cardiovascular systems by inhibiting vascular calcification, regulating mitochondrial function, and participating in antioxidant responses (45). B vitamins mainly participate in energy metabolism, DNA synthesis, and the maintenance of nervous system function, all of which are vital for cellular metabolism and nerve conduction (46, 47). In terms of immune regulation, the gut microbiota plays an important role as an immune barrier and regulator. It tightly binds to intestinal mucosal epithelial cells, forming a layer of physical and chemical biofilms that competitively exclude and prevent the adhesion and invasion of potential pathogens (48). The gut microbiota can also stimulate the development and maturation of gut-associated lymphoid tissue (GALT), promoting the differentiation and activity regulation of immune cells, thereby enhancing the body’s immunity (49, 50).

Moreover, the gut microbiota and its metabolites, such as SCFAs, lipopolysaccharide structures, and peptidoglycans, are essential stimulatory factors for the development and continuous maturation of GALT (51). They promote the differentiation, training, and functional regulation of immune cells, including dendritic cells, macrophages, T lymphocytes, and B lymphocytes. This process is crucial for maintaining local and systemic immune balance by inducing immune tolerance and regulated inflammatory responses (52). Specific strains of bifidobacteria can activate macrophages and regulate the activity of T lymphocytes, such as regulatory T cells (Treg) and T helper 17 cells (Th17), through their cell wall components or metabolites. This enhances the body’s immune surveillance and defense capabilities, thereby helping resist pathogen infections (29). The gut microbiota participates directly or indirectly in the synthesis and metabolism of various neurotransmitters, profoundly affecting nervous system function (53). Studies have found that the gut microbiota can synthesize neurotransmitters such as serotonin (5-HT) and dopamine (54). These neuroactive substances not only regulate intestinal motility, secretion, and sensation within the gut, mediated by the enteric nervous system, but can also influence CNS functions, including mood, cognition, and behavioral states. This occurs through various pathways of the gut microbiota–gut–brain axis, such as the vagus nerve, immune system, endocrine system, and SCFAs (15, 55). Increasing preclinical and clinical evidence suggests that dysbiosis of the gut microbiota disrupts the synthesis, metabolism, and signaling of neurotransmitters. Such disruptions are associated with an increased risk of mental disorders, including anxiety and depression (56, 57).

The gut microbiota is closely related to human health, and the stability of its composition and function is crucial for maintaining normal physiological processes and overall health (58). Once the gut microbiota is dysregulated, it may trigger a series of diseases, including digestive system disorders such as diarrhea, constipation, and inflammatory bowel disease; metabolic diseases such as obesity and diabetes; and neurological disorders such as anxiety, depression, and autism spectrum disorder (59, 60). Therefore, a deep understanding of the composition and function of the gut microbiota and its relationship with human health is of great significance for the prevention and treatment of related diseases.

### 2.2 SCI and gut microbiota dysbiosis

There is a close relationship between SCI and dysbiosis of the gut microbiota. SCI can trigger a series of physiological changes, leading to an imbalance of the gut microbiota, while dysbiosis of the gut microbiota can negatively affect the pathological process of SCI and the recovery of neurological function. After SCI, the gut microbiota undergoes significant changes in multiple aspects. In terms of microbial composition, the number of beneficial bacteria in the intestines of SCI patients is significantly reduced. For example, probiotics such as Bifidobacterium show a significant decline, while opportunistic pathogens such as Escherichia coli proliferate extensively (61). In an experiment on SCI rats, analysis using 16S rRNA sequencing technology found that the relative abundance of Firmicutes significantly decreased in the intestines of the rats after injury, while the relative abundance of Proteobacteria significantly increased. This trend is consistent with the changes in the gut microbiota of SCI patients observed in clinical studies (62, 63). In terms of microbial diversity, the diversity of the gut microbiota is also affected after SCI. A decrease in diversity indicates that the stability and function of the gut microbiota are compromised. Studies show that the Shannon index and Simpson index of diversity in the gut microbiota of SCI patients are lower than those of healthy individuals, indicating a reduction in the types and richness of the gut microbiota, leading to a simplification of the microbial structure (64). This reduction in microbial diversity may weaken the gut microbiota’s ability to adapt to environmental changes and resist pathogens, thereby increasing the risk of intestinal infections and diseases (65).

The causes of gut microbiota dysbiosis due to SCI are multifaceted, with autonomic nervous dysfunction being an important factor. After SCI, the patient’s autonomic nervous system function is impaired. This impairment affects the motility and secretory function of the intestine. The slowing of intestinal motility prolongs the transit time of food through the intestine (66). In addition, the abnormal secretory function of the intestine may lead to thinning of the intestinal mucus layer. Consequently, this damage compromises the intestinal mucosal barrier function, increasing susceptibility to pathogenic bacterial invasion (67). Meanwhile, the imbalance of immune regulation after SCI, the impairment of GALT function, and the decreased ability of immune cells to recognize and regulate intestinal microbiota exacerbate dysbiosis (68). Moreover, antibiotic use is also a common cause of intestinal microbiota dysbiosis (69). SCI patients are prone to infections, such as pulmonary infections and urinary tract infections, and often require antibiotic treatment (64). However, while antibiotics kill pathogenic bacteria, they also indiscriminately destroy beneficial bacteria in the gut, disrupting the balance of gut microbiota (70). Long-term or inappropriate use of antibiotics can cause irreversible changes in the structure and function of the gut microbiota and even induce small intestinal bacterial overgrowth (i.e., an abnormal increase in the number and/or changes in the composition of bacteria in the small intestinal lumen). This condition significantly increases the risk of gut dysbiosis and related complications (71). Changes in nutritional intake and metabolism can also impact gut microbiota. SCI patients may experience insufficient or unbalanced nutritional intake due to decreased appetite and digestive absorption dysfunction (72, 73). The lack of nutrients can affect the growth and metabolism of gut microbiota, limiting the nutritional support available to beneficial bacteria and thereby affecting the balance of gut microbiota (74).

## 3 The impact of gut microbiota dysbiosis on SCI

After SCI, the role of gut microbiota dysbiosis in multi-system pathological processes has received increasing attention. SCI can induce gut microbial imbalance by damaging autonomic nerve function and intestinal barrier integrity (75). Gut microbiota dysbiosis further triggers a series of pathological cascade reactions, including impaired intestinal barrier function, enhanced oxidative stress, abnormal immune response, and neurological dysfunction. The reduction of SCFAs and changes in bile acid metabolism disrupt intestinal homeostasis, leading to constipation, inflammation, and metabolic abnormalities. Oxidative stress and the accumulation of reactive oxygen species (ROS) can activate macrophages and the NLRP3 inflammasome, promoting neuroinflammation and inhibiting neuronal remodeling (76). Additionally, the decrease in 5-HT levels and the excessive activation of microglia jointly contribute to the development of depression and neuropathic pain. The translocation of microbiota and their metabolites into the bloodstream can induce systemic infections, further exacerbating neural and neuronal injury and the inflammatory response (77). Overall, gut microbiota dysbiosis after SCI is an important hub connecting local neural and neuronal injury with systemic metabolic-immune disorders, which lays the foundation for complications and disease progression.

### 3.1 Impaired intestinal barrier function

Neurogenic bowel dysfunction (NBD) is one of the common severe complications after SCI, significantly affecting the quality of life and rehabilitation process of patients (78, 79). There is a close and complex relationship between gut microbiota and NBD; the imbalance of gut microbiota plays a key role in the occurrence and development of NBD after SCI (80). Studies have found that beneficial bacteria, such as Bifidobacterium and Lactobacillus, significantly decrease in the intestines of SCI patients, while harmful bacteria, such as Escherichia coli and Enterococcus, overgrow (81). This dysbiosis can lead to impaired intestinal barrier function, increased intestinal permeability, and facilitated entry of bacteria and their metabolites into the bloodstream, triggering a localized inflammatory response (82). This inflammatory response further damages intestinal nerve and muscle function, exacerbating the symptoms of NBD and forming a vicious cycle (83). The metabolites of gut microbiota also participate in the pathological process of NBD. SCFAs are the main products of the fermentation of dietary fiber by gut microbiota. Under normal circumstances, SCFAs play an important role in maintaining normal intestinal function (84). However, in the case of gut microbiota dysbiosis after SCI, both the production and specific composition of SCFAs change, affecting intestinal motility and secretion function (85). Butyrate, as an important SCFAs, promotes intestinal motility and regulates the secretion of hormones by intestinal endocrine cells. After SCI, the number of butyrate-producing bacteria in the intestines decreases, leading to reduced butyrate levels, weakened intestinal motility, and defecation dysfunction (86).

NBD can also have an adverse effect on the gut microbiota. NBD leads to delayed intestinal emptying, causing stool to accumulate in the intestine, which alters the intestinal microenvironment, such as pH and oxidation-reduction potential (87). Consequently, these changes in the microenvironment can affect the growth, metabolism, and survival of the gut microbiota (88). Delayed intestinal emptying can also result in the accumulation of harmful substances and toxins in the intestine; these exert toxic effects on the gut microbiota and further disrupt its balance (89). Additionally, NBD can impair the immune functions of the intestinal mucosa, reducing the intestine’s resistance to pathogens and increasing the risk of intestinal infections, thereby leading to changes in the composition and functions of the gut microbiota (90, 91).

### 3.2 Inflammatory response

The inflammatory response plays a key role in the pathological process of SCI, while the gut microbiota exerts an important regulatory effect in this process (92, 93). After SCI, the body’s immune system is activated, triggering a series of complex inflammatory responses (94, 95). In the early stages of injury, there is a large infiltration of immune cells in the spinal cord, including neutrophils, macrophages, and lymphocytes (96). Once activated, these immune cells release various inflammatory cytokines, such as TNF-α, IL-1β, and IL-6 (97, 98). TNF-α can activate microglia and astrocytes that cause them to release more inflammatory mediators, such as nitric oxide and prostaglandin E2. These mediators further damage neural cells or neurons and inhibit their regeneration and repair (99). IL-1β and IL-6 can promote the infiltration and activation of inflammatory cells, exacerbating the inflammatory response at the SCI site and leading to worsening neurological dysfunction (100). Moreover, the inflammatory response also causes damage to the endothelial cells of blood vessels at the SCI site, affecting local blood circulation. This results in ischemia and hypoxia of neural cells or neurons, further aggravating neurological damage (101).

The dysbiosis of gut microbiota is closely related to the inflammatory response after SCI, with the microbial imbalance exacerbating inflammation through various pathways. The intestinal mucosal barrier is a crucial defense line against pathogen invasion, comprising physical, chemical, biological, and immune barriers (75, 102). Dysbiosis can impair intestinal barrier function, allowing bacteria and their metabolites in the gut to translocate into the bloodstream (103). Moreover, this imbalance leads to elevated levels of endotoxins, such as LPS, in the gut. LPS can enter the bloodstream through the damaged intestinal barrier, bind to TLR4 on the surface of host immune cells, and activate the nuclear factor-kappa B (NF-κB) signaling pathway, thereby inducing the release of pro-inflammatory cytokines (such as TNF-α, IL-1β, and IL-6), triggering a systemic inflammatory response (104). Endotoxins also activate the immune system, prompting immune cells to release large amounts of inflammatory cytokines. Once these cytokines enter the CNS, they can exacerbate neuroinflammation at the site of SCI, further damaging neural tissue (105, 106). The imbalance of gut microbiota can alter the activity and distribution of immune cells in the gut, leading to their overactivation (107). Studies have shown that dysbiosis causes functional abnormalities in immune cells such as T lymphocytes and B lymphocytes in the gut, resulting in increased release of inflammatory cytokines, such as TNF-α and IL-1β, thereby worsening inflammation after SCI (108). Additionally, gut microbial imbalance can influence the differentiation and development of immune cells, skewing them towards a pro-inflammatory phenotype and further aggravating the inflammatory response (109).

The metabolites of the gut microbiota also play an important role in the inflammatory response. SCFAs are the main metabolites of dietary fiber and other carbohydrates fermented by the gut microbiota, have significant anti-inflammatory effects (110). Butyrate can inhibit the activation of the NF-κB signaling pathway, thus reducing the production of inflammatory cytokines (111, 112). NF-κB is an important transcription factor that plays a core regulatory role in the inflammatory response, and its activation leads to increased expression of various inflammatory cytokines (113). By inhibiting the activation of NF-κB, butyrate lowers the levels of these inflammatory cytokines, thereby alleviating the inflammatory response at the SCI site (114). Acetate and propionate also exhibit notable anti-inflammatory and immunoregulatory effects. They modulate the activity of immune cells, promote the secretion of anti-inflammatory cytokines such as IL-10, and inhibit the excessive activation of the inflammatory response (115, 116). When the gut microbiota is dysregulated, decreased SCFAs production fails to suppress the inflammatory response effectively, resulting in its exacerbation (117, 118).

### 3.3 Oxidative stress

After SCI, the body undergoes oxidative stress caused by tissue ischemia and hypoxia resulting from SCI, leading to a series of redox imbalances and the production of large amounts of ROS and reactive nitrogen species (RNS) (119). These free radicals have high chemical reactivity and can damage biomacromolecules such as lipids, proteins, and DNA within cells, thereby affecting normal cellular functions and survival (120, 121). The gut microbiota plays an important regulatory role in oxidative stress, and its metabolites and compositional changes significantly influence the level of oxidative stress after SCI.

After SCI, the gut microbiota becomes dysregulated, characterized by a decrease in beneficial bacteria and an increase in harmful bacteria. The massive proliferation of harmful bacteria such as Escherichia coli and Staphylococcus aureus produces more ROS and RNS, exacerbating oxidative stress (122, 123). These harmful bacteria activate inflammatory cells by producing exotoxins, endotoxins, and other substances. Inflammatory cells then release inflammatory cytokines, which in turn induce oxidative stress (124). The LPS produced by Escherichia coli activates macrophages, causing them to release inflammatory cytokines such as TNF-α and IL-1β. These cytokines stimulate cells to produce more ROS and RNS, leading to elevated levels of oxidative stress (125). The reduction of beneficial bacteria such as Bifidobacterium and Lactobacillus weakens their inhibitory effect on oxidative stress. Bifidobacterium and Lactobacillus can directly scavenge ROS and RNS through the production of bacteriocins and SCFAs, which have anti-inflammatory properties rather than classical antioxidant activity. They also regulate immune responses to reduce the release of inflammatory cytokines, thereby alleviating oxidative stress (126, 127).

### 3.4 Neural function repair

The recovery process after SCI plays an important role in the prognosis of injury, while dysbiosis of gut microbiota following injury significantly impacts neural repair (128). TLRs are an important class of pattern recognition receptors that recognize pathogen-associated molecular patterns (PAMPs) and damage-associated molecular patterns (DAMPs). They activate immune cells and initiate the immune response (129, 130). After SCI, changes in gut microbiota can affect the expression and activation of TLRs; consequently, this influences neural remodeling (131). Among them, TLR4 is a well-studied receptor that recognizes bacterial LPS and other PAMPs (132). In SCI models, dysbiosis of gut microbiota leads to increased intestinal permeability, allowing LPS to enter the systemic circulation. This activates the TLR4 signaling pathway, triggers inflammatory responses and damages neural tissue (133, 134). Furthermore, the NLRP3 inflammasome is a multi-protein complex that detects intracellular danger signals, activates caspase-1, and promotes the maturation and release of inflammatory factors such as IL-1β and IL-18 (135). After SCI, imbalance of gut microbiota can lead to abnormal accumulation of metabolic products, such as LPS and imbalance of SCFAs, thereby stimulating excessive activation of the NLRP3 inflammasome. This triggers persistent inflammatory responses and inhibits neural remodeling (136). Animal experiments have shown that in SCI models, dysbiosis of gut microbiota significantly upregulates the expression levels of NLRP3, caspase-1, and IL-1β. This enhances neuroinflammatory responses, exacerbates tissue damage, and delays functional recovery (137). These studies indicate that dysbiosis of gut microbiota can affect the recovery of neural function after SCI.

### 3.5 Impact on SCI complications

The impact of gut microbiome dysbiosis extends far beyond local nerve repair disorders, with systemic effects permeating various complications following SCI. Autonomic nervous system dysfunction caused by SCI, prolonged bed rest, and impaired intestinal barrier makes the gut microbiome prone to long-term dysbiosis. This persistent microbial abnormality has been confirmed by clinical studies to be closely related to metabolic disorders, chronic pain, mood disorders, and increased infection risk (138, 139). In terms of metabolic diseases, patients with SCI often exhibit metabolic alterations in which gut microbiome dysbiosis plays an important role (140). After SCI, changes in the gut microbiome can affect the digestion and absorption of nutrients, leading to abnormal energy metabolism. Dysbiosis can reduce the production of SCFAs, which are not only an important energy source for intestinal epithelial cells, but also participate in regulating fat metabolism and insulin sensitivity (141). SCFAs regulate adipocyte differentiation and lipid metabolism by activating G protein-coupled receptor 43 (GPR43), thereby inhibiting fat accumulation (142, 143). In SCI patients, dysbiosis leads to decreased SCFAs levels, which may trigger insulin resistance, impair blood glucose regulation, and increase the risk of diabetes (144). The gut microbiome also influences bile acid metabolism, which plays a key role in fat digestion and absorption. Changes in the gut microbiome after SCI can lead to alterations in bile acid composition and metabolic pathways, affecting fat digestion and absorption and thus being associated with metabolic diseases such as obesity and hyperlipidemia (145, 146). Studies have shown that the abundance of bile acid metabolism-related bacteria in the intestines of SCI patients decreases, potentially leading to disruption of the enterohepatic circulation of bile acids and affecting lipid metabolism (147).

Neuropathic pain is a common complication in SCI patients, and dysbiosis of the gut microbiota plays a role in its occurrence and development (148, 149). The gut microbiota can influence pain signal transmission by regulating the synthesis and release of neurotransmitters (150). As mentioned earlier, altered gut microbiota leads to reduced synthesis of neurotransmitters such as 5-HT and γ-aminobutyric acid (GABA), both of which are important in pain modulation (151). Serotonin regulates pain signal transmission by binding to 5-HT receptors, producing analgesic effects (152, 153). GABA, an inhibitory neurotransmitter, decreases neuronal excitability and reduces pain signal transmission (154, 155). After SCI, dysbiosis of the gut microbiota results in decreased levels of serotonin and GABA. This reduction increases the excitability of pain pathways, thereby exacerbating neuropathic pain (156). Furthermore, gut microbiota imbalance can trigger inflammatory responses. The release of inflammatory factors sensitizes pain receptors, lowers the pain threshold, and increases patients’ sensitivity to pain (157, 158). In SCI patients, dysbiosis activates immune cells in the GALT. These immune cells release numerous inflammatory factors, such as TNF-α and IL-1β. These factors can enter the spinal cord via blood circulation or neural pathways; they activate microglia and astrocytes in the spinal cord, trigger neuroinflammatory responses, and further exacerbate neuropathic pain (118, 159, 160).

Depression is also a common psychological complication in SCI patients, and the imbalance of gut microbiota is closely related to its occurrence (161, 162). Gut microbiota can influence the neurochemistry and neurophysiological functions of the brain through various pathways, thereby affecting mood and psychological state (163, 164). Dysbiosis of gut microbiota can lead to neurotransmitter metabolism disorders, and the reduction of 5-HT, an important neurotransmitter for mood regulation, is closely associated with depression (165, 166). After SCI, changes in gut microbiota affect the metabolic pathways of tryptophan. Specifically, more tryptophan is metabolized into kynurenine, while less tryptophan is available for synthesizing 5-HT. This results in lower levels of 5-HT and increases the risk of depression (167–169). The imbalance of gut microbiota can also trigger inflammatory responses. Inflammatory factors can enter the brain through the blood-brain barrier (BBB), affecting the synthesis and release of neurotransmitters, regulating neuroplasticity and neuroendocrine functions, and leading to depressive symptoms (170–172). Studies have shown that in SCI patients, gut microbiota imbalance is positively correlated with elevated serum levels of inflammatory factors and the severity of depressive symptoms (64). Supplementing probiotics or prebiotics to restore gut microbiota balance can reduce inflammatory factor levels, improve neurotransmitter metabolism, and alleviate depressive symptoms (173, 174).

**Figure 2:**
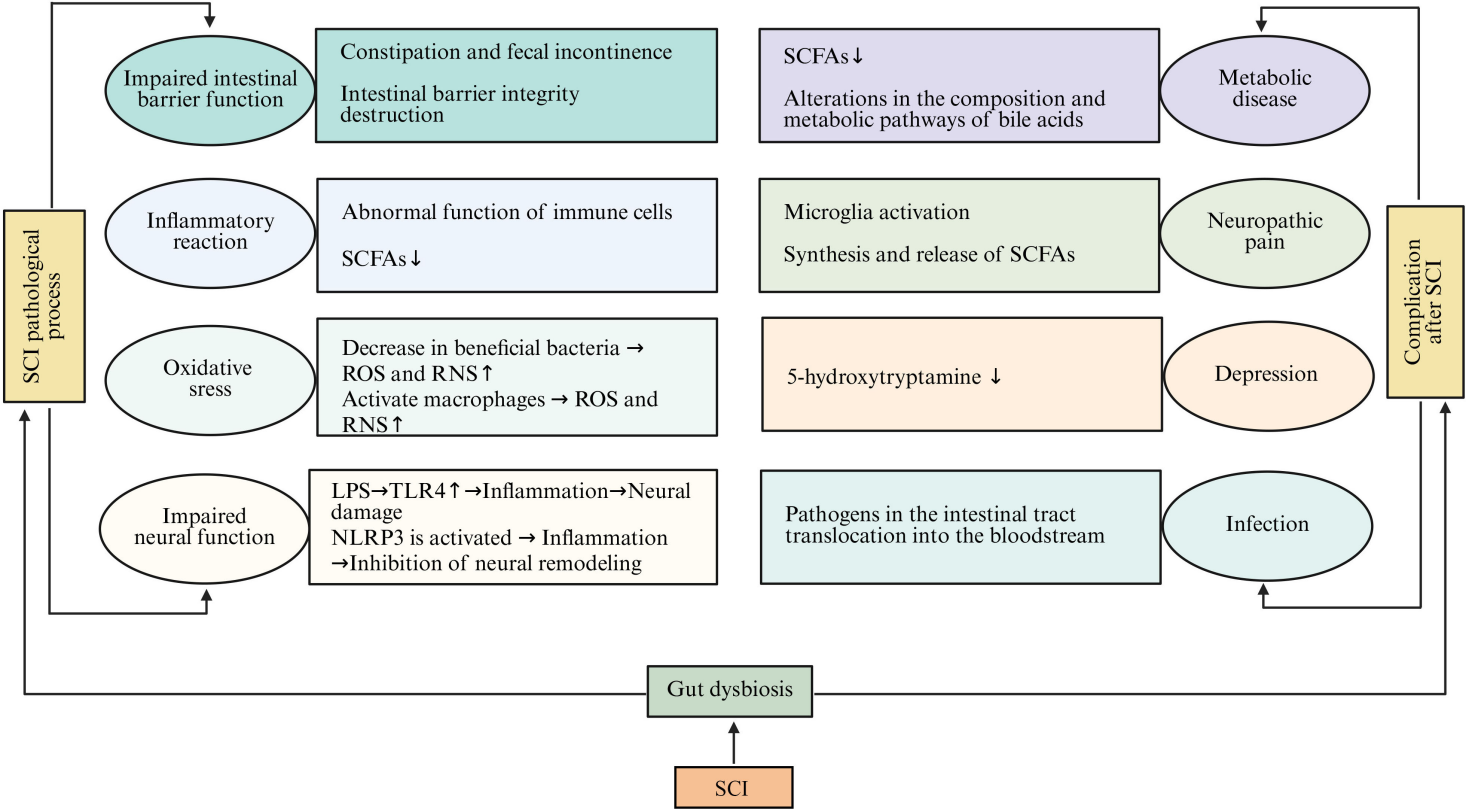
The mechanism of gut microbiota dysbiosis in the pathological processes of multiple systems and the formation of complications after SCI. SCI can lead to gut microbiota dysbiosis by damaging autonomic neural function and intestinal barrier integrity. Gut microbiota dysbiosis further triggers various pathological cascade reactions, including impaired intestinal barrier function, enhanced oxidative stress, abnormal immune response, and neurological dysfunction. The reduction of SCFAs and changes in bile acid metabolism cause an imbalance in intestinal homeostasis. This imbalance leads to constipation, inflammation, and metabolic abnormalities. Oxidative stress and the accumulation of ROS activate macrophages and the NLRP3 inflammasome, promoting neuroinflammation and inhibiting neural remodeling. Meanwhile, the decrease in serotonin levels and the excessive activation of microglia jointly contribute to the occurrence of depression and neuropathic pain. The translocation of gut microbiota and their metabolites into the bloodstream can induce systemic infections, which further exacerbate nerve injury and inflammatory responses. (SCI, Spinal cord injury; SCFAs, Short-chain fatty acids; ROS, Reactive oxygen species; RNS, Reactive nitrogen species; NLRP3, NLR family pyrin domain-containing 3; LPS, Lipopolysaccharide).

Infections are serious and frequent complications in SCI patients, with urinary tract infections and pulmonary infections being particularly prevalent. Gut microbiota imbalance increases the risk of these infections (138, 175). It also impairs immune system function, reducing the body’s ability to resist infections (176). Gut microbiota plays a crucial role in the development and regulation of the immune system, and its imbalance can lead to abnormal differentiation and function of immune cells, decreasing the body’s capacity to recognize and eliminate pathogens (177–179). Additionally, SCI patients are already prone to these infections due to prolonged bed rest, catheterization, and other factors, and gut microbiota imbalance further exacerbates this risk (180) (**Figure 2**).

In summary, gut function and gut microbiota are both involved in the pathophysiological process of SCI. SCI activates TLRs and the NLRP3 inflammasome; this upregulates the expression of neuroinflammatory factors and leads to neuroinflammation. In addition, SCI can cause autonomic nervous system dysfunction and intestinal immune imbalance. These changes weaken intestinal motility, increase intestinal barrier permeability, enhance intestinal protease activity, and raise the expression of pro-inflammatory cytokines in the gut. Consequently, these factors contribute to gut microbiota imbalance, characterized by a decrease in probiotics and an increase in LPS, produced by pathogenic bacteria. Harmful bacteria, along with their metabolites and cytokines, can cross the damaged intestinal barrier and blood-spinal cord barrier, potentially impairing neural function repair.

## 4 Mechanisms by which gut microbiota influences the pathogenesis of SCI

### 4.1 Mechanisms based on metabolic pathways

#### 4.1.1 Role of SCFAs

The production of SCFAs begins with substances such as dietary fibers entering the intestine. In the intestine, these substances are difficult to break down by the digestive enzymes secreted by the human body, thus becoming fermentation substrates for the gut microbiota (181). Anaerobic bacteria in the intestine, such as bifidobacteria and lactobacilli, utilize these substrates for fermentation and, through a series of complex enzymatic reactions, ultimately produce SCFAs (182). Most of these SCFAs are absorbed in the colon, enter the bloodstream, and subsequently affect various tissues and organs throughout the body.

SCFAs have shown significant neuroprotective effects after SCI, particularly butyrate and propionate (183). Butyrate, an important member of SCFAs, can precisely regulate gene expression by inhibiting the activity of histone deacetylases (HDACs) (184). In neural stem cells, butyrate inhibits HDACs, leading to the activation of certain genes related to the proliferation and differentiation of these cells. This promotes their differentiation into neurons and glial cells and increases the number of neural cells or neurons, thereby aiding in the repair of damaged nerves (114). Additionally, propionate has demonstrated neuroprotective effects in SCI, including inhibition of apoptosis, modulation of autophagy, anti-inflammation, anti-oxidation, and promotion of nerve regeneration (185). These effects may be achieved by mitigating mitochondrial dysfunction, reducing the expression of inflammatory cytokines, and regulating the levels of neurotrophic factors such as brain-derived neurotrophic factor (BDNF) and nerve growth factor (NGF).

Propionate, as another major SCFAs, can activate the NF-κB signaling pathway by binding to the GPR41/43 on the surface of macrophages and dendritic cells, thereby promoting the moderate secretion of TNF-α (186, 187). During the acute phase, the moderate release of TNF-α helps promote the recruitment of immune cells to the damaged area and the clearance of necrotic tissue. In the recovery phase, TNF-α also plays a role in the reconstruction of immune homeostasis by regulating the polarization of macrophages (from M1 to M2) and the balance of T-cell subsets-inhibiting Th1/Th17 and promoting Tregs/Th2 (188, 189). This biphasic regulation enables the propionate-TNF-α pathway to promote early inflammation clearance while driving later immune repair, thereby improving the neuroenvironment after SCI.

Moreover, acetate, propionate, and butyrate can generally regulate immune cell activity, inhibit the production of pro-inflammatory cytokines, and promote the secretion of anti-inflammatory cytokines (190). Specifically, butyrate can act on macrophages to inhibit their activation and reduce the release of pro-inflammatory cytokines such as TNF-α and IL-6. At the same time, it promotes the secretion of anti-inflammatory cytokines such as IL-10, thereby alleviating damage caused by inflammatory responses to neural tissue (191). SCFAs can also indirectly affect nervous system function by regulating hormone secretion from intestinal endocrine cells, which reduces inflammatory responses (192). Glucagon-like peptide-1 (GLP-1) is a hormone secreted by intestinal endocrine cells, and SCFAs can stimulate these cells to secrete GLP-1. This hormone can act on the CNS through the bloodstream, regulating neuroinflammatory responses and promoting neuroprotection (193).

SCFAs also play an important role in regulating immunity. SCFAs produced by the gut microbiota can regulate the development and function of GALT; promote the differentiation and regulation of immune cell activity; and enhance the body’s immunity (194). After SCI, immune function becomes dysregulated. In this context, SCFAs can restore immune balance by regulating immune cell function, thereby creating a favorable immune microenvironment for neural repair (195). Additionally, SCFAs promote the differentiation and proliferation of Tregs, which inhibit immune responses and reduce inflammation-induced damage to neural tissue (196, 197).

#### 4.1.2 Tryptophan metabolic pathway

Tryptophan is an essential amino acid for the human body; it undergoes a series of complex metabolic processes mediated by the gut microbiota (198). After being absorbed in the intestine, most tryptophan enters the liver for metabolism, while a small portion reaches the colon, where it is broken down by gut microbiota (199). In the colon, gut microbiota participates in tryptophan metabolism, producing various indole derivatives (200). Tryptophan metabolism mainly involves the kynurenine, 5-HT, and indole pathways (201). Among these, the kynurenine pathway is the primary route of tryptophan metabolism, degrading over 95% of tryptophan into various metabolites with immune regulatory and neuroactive properties. In this process, tryptophan is first converted into N-formylkynurenine by tryptophan-2,3-dioxygenase and indoleamine 2,3-dioxygenase 1 and 2 (IDO), then transformed into kynurenine by formamidase (202). Kynurenine and its downstream metabolites, such as quinolinic acid and kynurenic acid, modulate immune cell activity and influence neurotransmitter balance (203). In inflammatory states, upregulated IDO expression significantly enhances the kynurenine pathway, which decreases local tryptophan levels, thereby inhibiting T cell activation and proliferation and alleviating tissue damage caused by excessive immune responses (204). However, excessive accumulation of kynurenine metabolites may also cause immune dysfunction and exacerbate neuroinflammatory responses (205).

The 5-HT pathway is another important tryptophan metabolic pathway, where a small amount of tryptophan is catalyzed by tryptophan hydroxylase (TPH) to produce 5-hydroxytryptophan, which is further converted to 5-HT (206). 5-HT is not only an important neurotransmitter involved in regulating the balance of the intestinal environment, sleep, and gut-brain axis signaling, but also is a positive factor in neural repair after SCI (207). In the gut, 5-HT is mainly synthesized by tryptophan hydroxylase 1 in enterochromaffin cells, subsequently stored in platelets, and helps regulate physiological functions such as vascular contraction and the proliferation of vascular smooth muscle cells (208). However, 5-HT itself synthesized in the gut cannot cross the BBB to regulate CNS functions. The gut microbiota indirectly affects the synthesis of central 5-HT by regulating the availability of peripheral tryptophan, which serves as its precursor (209, 210). Tryptophan is an essential amino acid and a precursor for 5-HT synthesis. Its peripheral concentration is mainly controlled by dietary protein intake and the metabolic regulation of gut microbiota (211). The gut microbiota influences the proportion of tryptophan entering the brain by regulating its absorption, metabolism, and competitive transport against other neutral amino acids (212). Therefore, although gut-derived 5-HT itself cannot directly enter the CNS, the gut microbiota still shapes central serotonergic signaling pathways to some extent by regulating the systemic supply of tryptophan (213). Centrally synthesized 5-HT, as an important neurotransmitter, is involved in regulating various physiological processes such as mood, sleep, and appetite, and also plays a positive role in the recovery of neural function after SCI (214). After SCI, decreases in 5-HT levels can impair the transmission of neural signals, thereby influencing the recovery of neural function. Studies have found that the level of 5-HT in the serum of SCI patients is significantly reduced, and supplementing tryptophan or promoting the conversion of tryptophan to 5-HT can increase the level of 5-hydroxytryptamine, improving the patients’ neural function and emotional state (215). In terms of immune regulation, tryptophan metabolites exert effects through various pathways (216).

**Figure 3:**
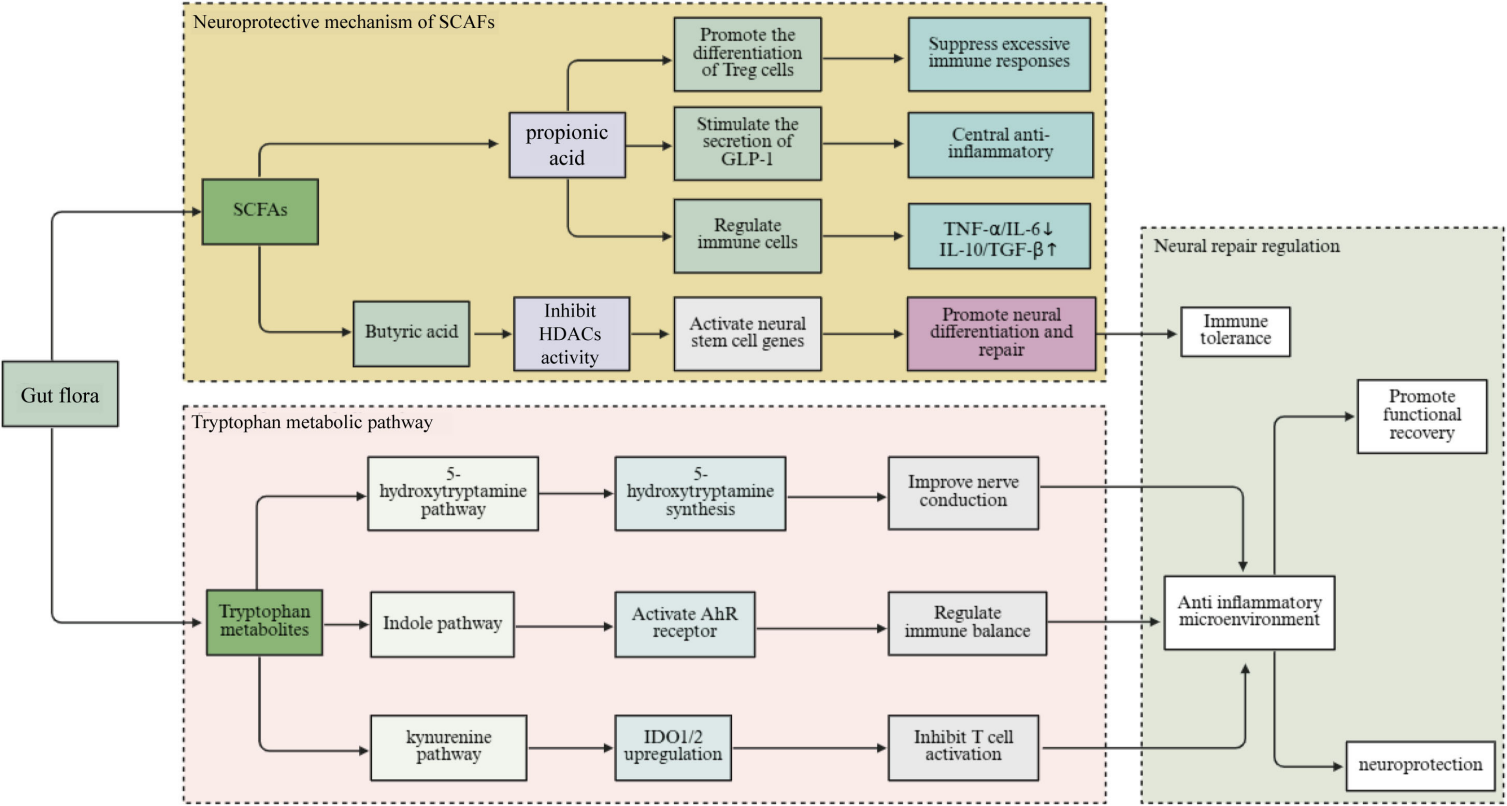
Neuroprotection by normal intestinal microbiota through metabolic pathways after SCI. It mainly promotes the recovery of neurological function after injury through the SCFAs pathway and the tryptophan metabolic pathway. (SCI, Spinal cord injury; HDACs, Histone deacetylases; SCFAs, Short-chain fatty acids; GLP-1, Glucagon-like peptide-1; AhR, Aryl hydrocarbon receptors; IDO1/2, Indoleamine 2, 3-dioxygenase 1 and 2; TNF-α, Tumor necrosis factor – α; IL-6, interleukin-6; IL-10, Interleukin-10; TGF-β, Transforming growth factor - β).

The pathway of indole and its derivatives is a metabolic pathway mediated by a distinctive enzyme system of gut bacteria, mainly involving Bacteroides, Clostridium, and Escherichia coli (217). Tryptophan is first converted into indole by bacterial enzymes. Subsequently, indole is further transformed into indole-3-acetic acid, indole-3-propionic acid, and other derivatives (218, 219). These indole metabolites can activate aryl hydrocarbon receptors (AhR) within host cells, thereby regulating immune responses and neuroinflammation (220). Following AhR activation, the secretion of anti-inflammatory cytokines (such as IL-10) can be induced, inhibiting the production of pro-inflammatory factors (such as TNF-α and IL-1β), and maintaining mucosal immune homeostasis (221, 222). In addition, indole derivatives provide a favorable immune microenvironment for neural repair by regulating the activity of astrocytes and microglia (223) (**Figure 3**).

### 4.2 Mechanisms based on immune pathways

#### 4.2.1 Regulation of cytokines

After SCI, there is a close and complex relationship between the imbalance of gut microbiota and the disruption of cytokine homeostasis; this interaction profoundly affects neural cells or neurons and the inflammatory response. SCI often leads to significant changes in the structure and function of gut microbiota, such as reduced microbial diversity, decreased beneficial bacteria, and overgrowth of pathogenic bacteria. This imbalance can cause damage to the gut barrier function, increasing gut permeability and allowing bacteria and their metabolites to translocate into the bloodstream. These translocated substances can activate the immune system, stimulating immune cells to secrete a large number of cytokines, thereby disrupting cytokine balance (224–226).

Cytokines, as key signaling molecules in the immune system, play a dual role in neuroinflammation and neural repair after SCI (227). On one hand, pro-inflammatory cytokines have a negative effect in neuroinflammation and neural repair after SCI. TNF-α is an important pro-inflammatory cytokine that can activate microglia and astrocytes, leading to the release of more inflammatory mediators, further exacerbating neuroinflammation (228). TNF-α can also induce neuronal apoptosis by activating the caspase family and other apoptosis-related proteins, promoting programmed cell death in neurons, which results in a decrease in the number of neurons and affects the recovery of neural function (229). IL-6 also has a strong pro-inflammatory effect; it can promote the activation and proliferation of immune cells, attracting more immune cells to infiltrate the injury site, worsening the inflammatory response (230). IL-6 can also amplify the inflammatory response by regulating the expression of other cytokines, forming an inflammatory cascade (231). Conversely, anti-inflammatory cytokines play a positive role in combating inflammation and promoting neural repair (232). IL-10 is a typical anti-inflammatory cytokine that can inhibit the production of pro-inflammatory cytokines by binding to receptors on the surface of immune cells and suppressing the activation of related signaling pathways. This reduces the secretion of pro-inflammatory cytokines such as TNF-α and IL-6 (233). IL-10 can also promote the polarization of immune cells toward an anti-inflammatory phenotype, regulating the intensity of the immune response and creating a relatively stable microenvironment for neural repair (234). TGF-β also has anti-inflammatory and neural repair-promoting effects; it can inhibit the activity of inflammatory cells, reduce the release of inflammatory mediators, and promote the proliferation and differentiation of neural stem cells, which contributes to the regeneration of damaged neural tissue (235).

In the pathological process after SCI, the equilibrium between pro-inflammatory cytokines and anti-inflammatory cytokines is crucial (236). When the expression of pro-inflammatory cytokines significantly exceeds that of anti-inflammatory cytokines, neuroinflammation worsens, leading to increased damage and apoptosis of neural cells or neurons, which severely impedes the recovery of nerve function (237). Conversely, when anti-inflammatory cytokines effectively inhibit pro-inflammatory cytokines and maintain cytokine homeostasis, the inflammatory response can be controlled, reducing nerve cell damage, improving the microenvironment for nerve repair, and facilitating functional recovery (232). Studies in animal models of SCI have shown that regulating the gut microbiota-by increasing the abundance of beneficial bacteria and reducing the overgrowth of harmful bacteria—can lower pro-inflammatory cytokine levels while enhancing the expression of anti-inflammatory cytokines, such as IL-10 and TGF-β. This modulation alleviates neuroinflammation and promotes nerve function recovery (238). These findings further demonstrate that gut microbiota imbalance plays a significant role in SCI pathogenesis by influencing cytokine homeostasis.

#### 4.2.2 TLRs and NLRP3 inflammasome

TLRs and NLRP3 inflammasomes play a key role in the spinal cord inflammation induced by dysbiosis of the gut microbiota, and their activation mechanisms are complex and interconnected (239). TLRs are important pattern recognition receptors that are widely expressed on the surface of immune cells and non-immune cells. Their activation mechanisms mainly involve recognizing PAMPs and DAMPs to initiate immune responses (240). In the case of dysbiosis of the gut microbiota after SCI, the intestinal barrier is impaired, allowing bacteria and their metabolites to enter the bloodstream. These substances contain PAMPs, such as LPS and peptidoglycan, which can specifically bind to TLRs (17); for example, TLR4 has a high affinity for LPS. When LPS enters the body, it forms a complex with TLR4 and its co-receptor myeloid differentiation factor 2. The formation of this complex recruits myeloid differentiation primary response protein 88 (MyD88). This recruitment activates mitogen-activated protein kinase (MAPKs) and NF-κB signaling pathways (241). The activation of the MAPKs signaling pathway leads to the upregulation of a series of inflammation-related genes, promoting the synthesis and release of inflammatory cytokines. NF-κB translocates from the cytoplasm to the nucleus. It then binds to the promoter regions of relevant genes and initiates the transcription and translation processes of inflammatory factors such as TNF-α and IL-6 (242, 243).

The NLRP3 inflammasome is a multiprotein complex mainly composed of NLRP3, apoptosis-associated speck-like protein, and caspase-1. Its activation mechanism is quite complex and usually requires the synergistic action of two distinct signals (244, 245). The first signal is the “priming signal,” which is primarily initiated via signaling pathways mediated by pattern recognition receptors such as TLRs, leading to the expression and synthesis of inflammasome-related proteins such as NLRP3 and pro-caspase-1 (246). In myelitis caused by gut microbiota dysbiosis, the TLR signaling pathway activated by the translocation of gut bacteria and their metabolites can provide this priming signal (17). The second signal is the “activation signal,” which can be provided by various stimulating factors, including potassium ion efflux, mitochondrial dysfunction-induced ROS production, and cathepsin B release from lysosomal damage (247, 248). Previous studies have shown that the NLRP3 complex is activated in the SCI mouse model, and pharmacological inhibition of the NLRP3 inflammasome has been confirmed to control neuroinflammation and reduce the severity of SCI (249). Additionally, the selective inhibitor of the NLRP3 inflammasome, MCC950, has been shown to regulate gut microbiota and effectively alleviate corticospinal tract injury after intracerebral hemorrhage (250). Transplantation of fecal microbiota from NLRP3 knockout mice can effectively alleviate astrocyte dysfunction in mice and improve depressive-like behavior induced by chronic unpredictable stress (251). Therefore, NLRP3 may serve as a bridge between gut microbiota and CNS diseases.

**Figure 4:**
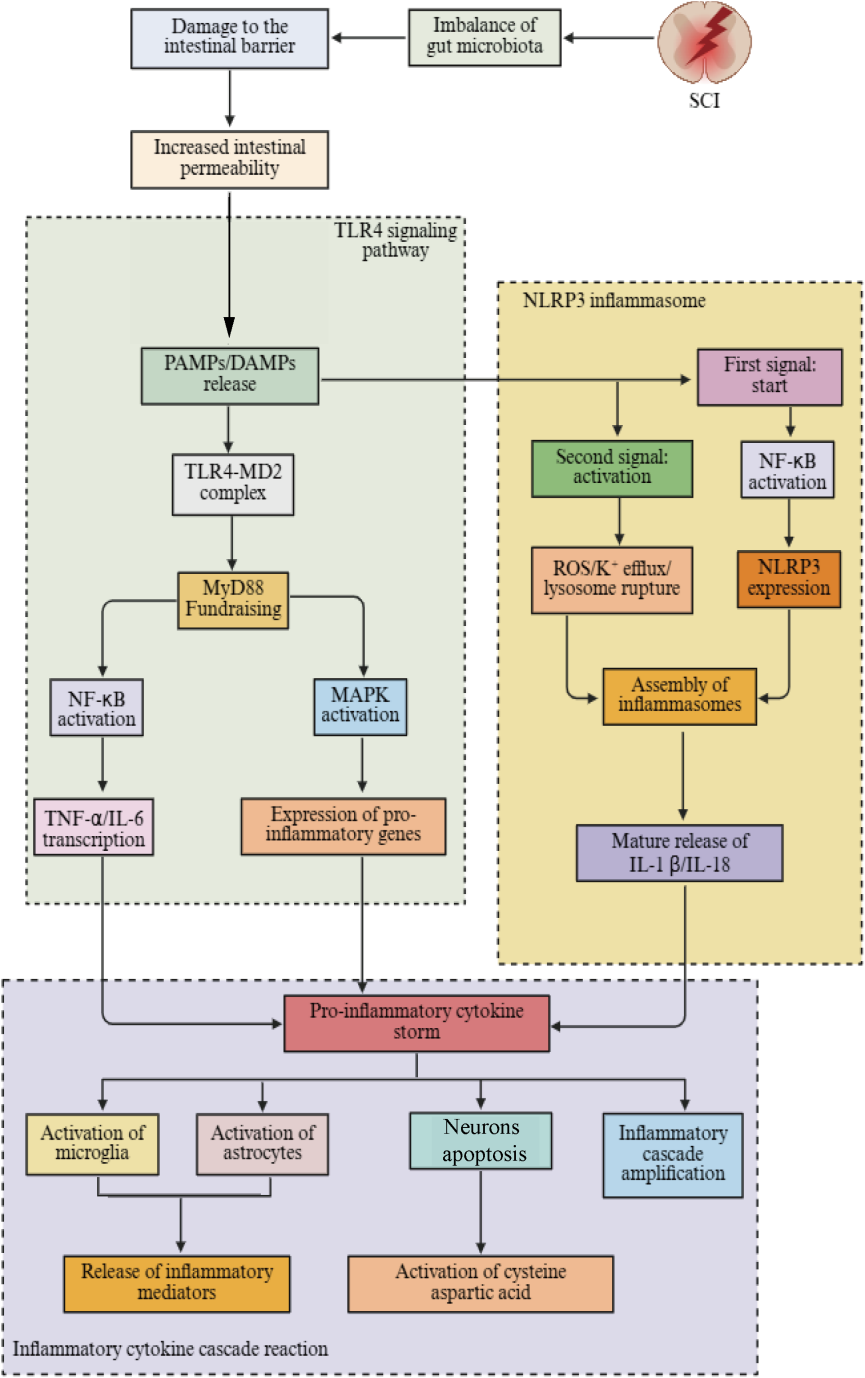
The impact of gut microbiota dysbiosis on nerve function through immune pathways after injury. Gut microbiota dysbiosis mainly influences SCI through cytokines, toll-like receptors, and the NLRP3 inflammasome. (SCI, Spinal cord injury; PAMPs, Pathogen-associated molecular patterns; DAMPs, Damage associated molecular patterns; TLR4, Toll-like receports-4; NF-κB, Nuclear factor-κB; TNF-α, Tumor necrosis factor – α; IL-18, Interleukin-18; IL-6, Interleukin-6; ROS, Reactive oxygen species; IL-1β, Interleukin-1β; TGF-β, Transforming growth factor-β; NLRP3, NLR family pyrin domain-containing-3; MAPK, Mitogen-activated protein kinase; MD2, Myeloid differentiation factor-2; MyD88, Myeloid differentiation primary response 88).

In spinal inflammation induced by gut microbiota dysbiosis, TLRs and the NLRP3 inflammasome interact to jointly promote the initiation and progression of inflammation. The inflammatory factors produced after TLRs activation, such as TNF-α and IL-6, not only directly participate in the inflammatory response but also serve as the first signal for the activation of the NLRP3 inflammasome, promoting its assembly and subsequent activation. Subsequently, caspase-1 is activated by the NLRP3 inflammasome, which cleaves and matures key pro-inflammatory factors such as IL-1β and IL-18, thereby amplifying inflammatory signals and exacerbating spinal neuroinflammation (252). Supporting these mechanisms, studies have shown that in animal models of SCI, inhibiting TLRs signaling pathways or the activation of the NLRP3 inflammasome can significantly alleviate spinal inflammation, reduce neural and neuronal cell damage, and promote the recovery of neurological function (253, 254) (**Figure 4**). This clearly demonstrates the important role of TLRs and the NLRP3 inflammasome in spinal inflammation induced by gut microbiota dysbiosis and provides potential targets for the treatment of SCI (**Table 1**).

**Table 1:** The role of key metabolic and immune signaling pathways of gut microbiota in neuroinflammation and repair after SCI.

Pathway type	Key molecules/ metabolites	Main receptor/ targets	Main signaling mechanisms	Effects on neuroinflamm-ation	Effects on neural repair
SCFAs pathway	Acetic acid, propionic acid, butyric acid	GPR41, GPR43, HDACs	Produced by the fermentation of dietary fiber by anaerobic bacteria; butyrates inhibit HDACs to regulate gene expression; propionate activates NF-κB through GPR41/43, promotes GLP-1 secretion.	Inhibit pro-inflammatory factors such as TNF-α and IL-6, and promote anti-inflammatory signals such as IL-10 and TGF-β.	Promote the proliferation and differentiation of neural stem cells, upregulate BDNF/NGF, and enhance neural plasticity.
Tryptophan metabolic pathway	Kynurenine, indole derivatives, 5-hydroxytryptamine	AhR, TPH1, TPH2, IDO1/2	Metabolic regulation of immune homeostasis and neural signals through pathways such as IDO1/2, AhR, and TPH1	Kynurenine pathway inhibits T cell activation; Indole-AhR promotes anti-inflammatory; Serotonin improves mood and inflammatory response.	Promote the balance of neurotransmitters and signal transduction, and enhance nerve repair.
TLR4 signaling pathway	LPS	TLR4/ NF-κB/ MAPK	After the destruction of the intestinal barrier, LPS activates TLR4, inducing the upregulation of pro-inflammatory gene expression of NF-κB and MAPK.	Microglia and astrocytes were activated, and TNF-α, IL-1β and IL-6 were upregulated.	Inflammation inhibits neural remodeling and myelin formation, leading to neuronal apoptosis.
NLRP3 inflammasome pathway	NLRP3, Caspase-1, IL-1β, IL-18	NLRP3 inflammasome	Two-step activation: ① TLR4-NF-κB signaling initiation ② ROS, K^+^ efflux, lysosomal damage activate NLRP3 complex.	Promote the maturation and release of IL-1β/IL-18, exacerbating neuroinflammation.	Inhibiting NLRP3 or Caspase-1 can promote axonal regeneration and functional recovery.
Comprehensive action axis	SCFAs–TLR4–NLRP3–AhR interaction pathway	HDACs, GPR43, NF-κB, AhR	SCFAs inhibit NF-κB/NLRP3; indole–AhR activates IL-10; LPS–TLR4 triggers pro-inflammatory chain, forming dynamic balance.	SCFAs and AhR signaling dominate, inflammation is controlled; when TLR4–NLRP3 is excessively activated, inflammation persists.	SCFAs and tryptophan metabolites promote regeneration by regulating immune homeostasis and neurotrophic factors.

## 5 Treatment methods for SCI based on gut microbiota

### 5.1 Fecal microbiota transplantation

Fecal Microbiota Transplantation (FMT) is an innovative method for treating diseases by reconstructing the gut microbiota. It has been studied as a potential therapy for inflammatory bowel diseases and Clostridioides difficile infections (255). In the field of SCI treatment, FMT has demonstrated promising therapeutic potential. Multiple animal experiments have provided strong evidence supporting the role of FMT in SCI treatment. After transplanting fecal microbiota from healthy uninjured mice into SCI mice, researchers observed a series of positive effects. Neurologically, SCI mice receiving FMT showed significant functional recovery, including notable improvements in exercise capacity and coordination. In these experiments, specific behavioral tests, such as the Basso-Beattie-Bresnahan locomotor rating scale (BBB score), visually demonstrated improvements in the hind limb motor function of the mice, with their hind limb movements becoming more agile and capable of performing more complex actions (256, 257). Additionally, FMT can promote neuronal axon regeneration. Histological analysis revealed that the number and length of neuronal axons in the spinal cord tissue of mice receiving FMT increased, indicating that FMT provides a favorable environment for neuronal growth and repair, thereby facilitating neural signal conduction (258). FMT also plays an important role in improving animal body weight and metabolic characteristics. SCI mice often experience weight loss and metabolic disorders; however, after receiving FMT, the mice gradually restored their weight to normal, and metabolic indicators stabilized (258). The integrity of the intestinal barrier and gastrointestinal motility were also enhanced. This was evidenced by increased expression of intestinal tight junction proteins and reduced intestinal permeability, which effectively prevented the translocation of bacteria and their metabolites and reduced the occurrence of inflammatory responses (259). Furthermore, FMT has shown significant therapeutic advantages for SCI-related complications, such as depression and anxiety symptoms (19).

However, FMT also faces some challenges in the treatment of SCI (260). Among them, safety issues are the primary focus of concern. Despite strict measures taken during donor screening and stool processing, there remains a risk of infection by pathogens such as bacteria, viruses, and parasites, which may lead to severe infectious complications in patients (261). In addition to safety concerns, the standardization and harmonization of FMT are urgent issues to be addressed. Currently, there are no unified standards for the operation process, donor selection criteria, transplant dose, and frequency of FMT. This lack of standardization leads to significant differences in various studies and clinical practices, complicating the promotion and application of FMT (262). Individual differences in the effects of FMT cannot be ignored, as the gut microbiota environment, immune system, and underlying health conditions vary among different patients, resulting in variable responses to FMT. Some patients may experience poor outcomes or even adverse reactions following FMT (263). Therefore, future research should focus on the standardization of donor selection criteria, precise individual matching strategies, and long-term follow-up to ensure safety and stable efficacy.

### 5.2 Probiotic intervention

Probiotics are a class of beneficial live microorganisms for the host that can positively impact human health by regulating the balance of gut microbiota. Common types of probiotics include diverse genera such as Bifidobacterium, Lactobacillus, and yeast species (264, 265). Bifidobacterium, one of the most widely used probiotics, plays a key role in maintaining normal intestinal function. It regulates the balance of gut microbiota by competing with harmful bacteria for nutrients and adhesion sites, inhibiting the growth of pathogenic bacteria, and promoting the proliferation of beneficial bacteria (266). Additionally, Bifidobacterium can enhance the intestinal mucosal barrier function. Its cell wall components and metabolites stimulate the proliferation and differentiation of intestinal mucosal cells and increase mucus secretion. These effects strengthen the intestinal mucosal barrier, prevent the invasion of harmful substances, and promote immune system function by activating immune cells and enhancing immune defenses (267).

Lactobacillus is another common probiotic that secretes lactic acid and other beneficial substances to regulate the intestinal environment (268). The lactic acid produced by Lactobacillus lowers the intestinal pH, creating an acidic environment unfavorable for the growth of harmful bacteria, thereby inhibiting them (269). Lactobacillus also enhances intestinal immunity, as its cell components and metabolites stimulate the activity of intestinal immune cells, strengthening the immune defense function of the intestine (270). Furthermore, Lactobacillus can improve constipation, promote intestinal peristalsis, and facilitate the expulsion of feces from the body (271). The yeast Saccharomyces boulardii, in addition to regulating gut balance, can improve the body’s nutrient utilization efficiency. It produces various enzymes that help break down complex food components, promoting nutrient absorption. Saccharomyces boulardii also inhibits the growth of harmful bacteria, maintaining the stability of the gut microecology (272).

In the treatment of SCI, probiotic intervention plays an important role in the therapeutic mechanism. Probiotics can regulate the structure of gut microbiota, increase the abundance of beneficial bacteria, and inhibit the growth of harmful bacteria, thereby restoring gut microbiota balance (273, 274). After SCI, gut microbiota dysbiosis occurs, characterized by a decrease in beneficial bacteria and an increase in harmful bacteria, while supplementing with probiotics can improve this condition (275). Research shows that after probiotic intervention in SCI mice, the number of beneficial bacteria, such as Bifidobacterium and Lactobacillus, significantly increases, while the number of harmful bacteria, such as Escherichia coli and Clostridium, significantly decreases (276). Probiotics indirectly affect nervous system function by regulating the secretion of hormones from intestinal endocrine cells and promoting the recovery of neural function (277).

However, when applying probiotic intervention in SCI treatment, several considerations need to be addressed. Different types of probiotics have varying regulatory effects on gut microbiota, so it is necessary to select appropriate probiotic species and strains based on the specific conditions of the patient. Attention should be paid to the dosage and administration method of probiotics. A dosage that is too low may not achieve the expected therapeutic effect, while high doses may lead to excessive competition in the gut microbiota, bloating, diarrhea, and even bacteremia or infectious complications in individuals with weakened immune function (278, 279). At the same time, the storage and transportation conditions of probiotics can also affect their activity, so they need to be stored and used strictly according to requirements (280).

### 5.3 Dietary intervention

Diet, as one of the key factors influencing the gut microbiome, has a profound impact on the composition, structure and functions of the gut microbiome. Different dietary components can selectively promote or inhibit the growth of specific types of gut microbes, thereby altering the overall composition of the gut microbiome (281). A study indicates that a high-fat and high-sugar diet after SCI hinders the recovery of sensory-motor and bladder functions, reduces the capacity for nerve regeneration, enhances microglial proliferation, and exacerbates the loss of oligodendrocytes (282). In contrast, a high-fiber diet positively promotes the balance and diversity of the gut microbiome. Cellulose, a polysaccharide indigestible by human enzymes, can serve as a high-quality food source for beneficial bacteria (283). In the gut, beneficial bacteria such as bifidobacteria and lactobacilli can utilize cellulose through fermentation, obtaining the energy needed for growth and thus proliferating in large numbers (284). A high-fiber diet can also increase undigested food residues in the gut and promote intestinal peristalsis, which facilitates the smooth expulsion of intestinal contents. This reduces the retention time of harmful bacteria in the gut and creates a more favorable living environment for beneficial bacteria (285, 286). Studies have shown that populations with habitual high dietary fiber intake have significantly increased numbers of bifidobacteria and lactobacilli in their intestines, while the proportion of harmful bacteria such as Escherichia coli and Clostridium perfringens is relatively reduced (287, 288).

In addition to dietary fibers, prebiotics play a crucial role in modulating the gut microbiome. Prebiotics are substances that can selectively stimulate the growth and activity of beneficial bacteria in the gut. Common prebiotics include fructooligosaccharides, xylooligosaccharides, and inulin (283). They cannot be digested and absorbed by the human body but serve as substrates for beneficial gut bacteria, promoting their growth and reproduction (289). Fructooligosaccharides can be fermented and metabolized by beneficial bacteria such as bifidobacteria and lactobacilli, producing SCFAs like acetate, propionate, and butyrate as metabolic end-products (290). These SCFAs not only provide energy for intestinal epithelial cells, promoting the growth and repair of the intestinal mucosa, but also regulate gut immune functions and inhibit the growth of harmful bacteria (291). Research has shown that supplementation with prebiotics can enhance gut barrier function and reduce inflammation levels (292). Therefore, a comprehensive nutritional strategy based on optimizing dietary structure and supplementing with prebiotics may become an important direction for future microbiome interventions in SCI.

### 5.4 Rehabilitation training combined with gut microbiota regulation

Rehabilitation training has a significant impact on gut microbiota, and there is a close synergistic mechanism between the two. Multiple meta-analyses indicate that rehabilitation training is an effective treatment for SCI (293, 294). Rehabilitation training regulates the structure and function of gut microbiota through various pathways. As an important component of rehabilitation training, exercise can increase intestinal blood flow and enhance nutrient availability for gut microbiota, thereby promoting the growth and diversification of the flora. Exercise also promotes intestinal peristalsis, accelerates the movement of intestinal contents, reduces the retention time of harmful bacteria, and fosters an environment conducive to beneficial bacterial growth (295, 296). Relevant studies have shown that long-term adherence to aerobic exercise, such as running and swimming, can increase the abundance of beneficial bacteria like lactic acid bacteria and Bifidobacterium in the gut while reducing the abundance of harmful bacteria such as Clostridium and Bacteroides fragilis (297). In a study involving mice, after a period of running training, the diversity of the gut microbiota significantly increased, the number of beneficial bacteria rose markedly, and the number of harmful bacteria decreased (298). These findings indicate that the exercise component of rehabilitation training can directly influence the intestinal microecological environment, altering the composition of gut microbiota.

Rehabilitation training combined with gut microbiota regulation exhibits significant synergistic effects. On one hand, the balance of gut microbiota plays an important supportive role in the effectiveness of rehabilitation interventions. The gut microbiota regulates immune functions, enhances immune function, and provides a solid physical foundation for rehabilitation (176). Metabolites such as SCFAs produced by the gut microbiota can provide energy for intestinal epithelial cells; promote the growth and repair of the intestinal mucosa; maintain the integrity of the intestinal barrier; and reduce the occurrence of inflammatory reactions. Together, these effects create a favorable internal environment for rehabilitation. The gut microbiota also participates in the synthesis and metabolism of neurotransmitters, affecting nervous system functions, which helps improve neural regulatory capacity during rehabilitation and promotes the recovery of neurological function (299, 300). On the other hand, rehabilitative exercise can further optimize the structure and function of the gut microbiota. Changes in the gut microbiota caused by exercise increase the abundance of beneficial bacteria. These bacteria promote the metabolism and utilization of nutrients by the gut microbiota, producing more beneficial metabolites for the body, such as SCFAs and B vitamins. These metabolites are not only beneficial for gut health, but can also influence systemic physiological functions through the bloodstream, further enhancing the effects of rehabilitation. Moreover, rehabilitative exercise can indirectly affect the growth and metabolism of gut microbiota by regulating the secretion of hormones from intestinal endocrine cells, forming a positive feedback loop that enhances rehabilitation outcomes (301) (**Table 2**).

**Table 2:** Application of gut microbiota intervention strategies in SCI treatment.

Intervention strategy	Specific method	Mechanism of action	Challenges	References
Fecal Microbiota Transplantation Transplanting	Transplant fecal microbiota from a healthy donor into the patient’s intestine	Reconstructing intestinal microbiota balance, promoting nerve repair, improving metabolism and intestinal barrier function	Potential pathogen infection, significant individual efficacy differences	(258, 259)
Probiotic intervention	Supplementing active probiotics (Bifidobacterium, Lactobacillus, Saccharomyces boulardii, etc.)	Regulating microbiota balance, enhancing intestinal barrier, inhibiting harmful bacteria, promoting immune and nerve function	Difficulty in dosage control, activity easily affected by transportation/storage	(267, 270, 272)
Dietary intervention	Supplementing high-fiber diet and prebiotics	Regulating microbiota composition through nutritional substrates, promoting the growth of probiotics	Need to maintain balance long-term, significant adaptability differences	(287, 288, 291)
Rehabilitation training + Microbiota regulation	Aerobic exercise (running, swimming, etc.) combined with microbiota intervention	Exercise improves intestinal environment: increases blood flow, promotes peristalsis, Microbiota supports training effects: immune regulation, nerve repair	Need to maintain balance long-term, significant adaptability differences	(299–301)

## 6 Analysis of current research status and future prospects

### 6.1 Existing research issues and challenges

Although there has been some progress in the research on the relationship between the gut microbiota and SCI, many issues and challenges still limit further development and clinical application in this field. Regarding the depth of mechanistic research, although it is now clear that the gut microbiota and its metabolites play a role in the pathogenic mechanism of SCI, the specific molecular mechanisms and signaling pathways are not yet fully understood. For example, in the regulatory mechanism of SCFAs on the proliferation and differentiation of neural stem cells, it is known that butyrate can inhibit the activity of HDACs; however, the specific gene targets downstream of HDACs and how these genes work together to promote the differentiation of neural stem cells still require in-depth research (302). In the tryptophan metabolic pathway, after tryptophan metabolites activate aryl hydrocarbon receptors, the mechanisms by which they regulate immune responses and neuroinflammation through complex signaling networks also need further exploration (303). Moreover, current research has not clarified which specific gut bacterial strains undergo persistent changes after SCI. Whether these changes are the “cause” or “result” of the pathological process of SCI remains controversial (304). For example, some reports indicate that changes in the ratio of Firmicutes to Bacteroidetes are associated with the degree of inflammation; however, there is a lack of longitudinal studies and causal validation (305). This issue reflects the complexity of the interaction between the gut microbiota and SCI, involving multiple systems and levels. Existing research often focuses on single factors, lacking an integrated understanding of the overall network.

In addition, when translating animal experimental results to human SCI patients, researchers also face significant limitations and contradictions. Currently, most studies are based on mouse or rat models, which differ from humans in intestinal anatomy, microbial composition, and immune and metabolic responses. The effects of probiotics or FMT observed in animal models do not always translate to clinical settings, and some studies even report contrary results, suggesting that differences between animal models and humans may lead to biased conclusions (306). Moreover, experimental animals are usually raised under sterile or standardized conditions, lacking the complex dietary, drug, and environmental exposure factors present in humans, which further limits the extrapolation of research results to clinical settings (307). Therefore, future research urgently needs to bridge the gap between experiments and clinical practice through a multi-species validation, organoid models, and clinical cohort studies.

Standardization and normalization of treatment methods are also important challenges faced by current research. Taking FMT as an example, there is a current lack of unified standards in donor screening criteria; fecal material processing techniques; transplantation routes; doses; and frequency for SCI treatment. The differences in protocols adopted by different studies are significant, making it difficult to compare and validate research results. This hinders the promotion and application of FMT in SCI treatment. Similarly, there is a lack of standardized protocols for probiotic and dietary interventions. Different types of probiotics exert varying effects on the gut microbiota and the efficacy of SCI treatment. Currently, no clear standards exist for selecting appropriate probiotic species and strains, nor for determining optimal doses and treatment durations. In dietary intervention, the impact of different dietary components on the gut microbiota is complex and diverse. Developing personalized dietary plans based on patients specific conditions requires further research and exploration. In addition, the lack of long-term safety assessments is a problem that cannot be ignored. Currently, SCI treatment methods targeting the gut microbiota are mostly in the stages of basic research and clinical trials, and there is relatively little data from long-term safety assessments. Although FMT has shown certain therapeutic effects in short-term experiments, there may be risks of pathogen infection and immune response activation in the long term. Probiotic intervention may lead to excessive regulation of the gut microbial ecology, causing new dysbiosis; moreover, long-term use of certain probiotics may also lead to antimicrobial resistance concerns (308, 309). If dietary intervention is not properly designed, it may affect the nutritional status of patients and lead to other health problems. Due to the lack of long-term follow-up studies, these potential safety hazards are difficult to comprehensively assess and monitor, which limits the translation of treatment methods from laboratory research to clinical application.

### 6.2 Future research directions

In the future, research in the field of gut microbiota and SCI should focus on in-depth mechanism studies, developing precise treatment plans, and exploring combination therapy strategies. In terms of in-depth mechanism research, multi-omics technologies should be employed, including metagenomics, transcriptomics, proteomics, and metabolomics to comprehensively analyze the molecular mechanisms and signaling pathways underlying the interactions between gut microbiota and SCI. Metagenomics technology should be used to conduct a detailed analysis of the gene composition and functions of gut microbiota to reveal the specific roles of different microbial communities in the pathogenesis of SCI. Using transcriptomics and proteomics technologies, the expression changes of gut microbiota-related genes and proteins after SCI should be studied; additionally, the impacts of these changes on neuronal function and the inflammatory response should be investigated. With the help of metabolomics technology, changes in the metabolites of gut microbiota should be analyzed to clarify their mechanisms in neural repair and inflammation regulation. Moreover, attention should be paid to the dynamic changes in gut microbiota following SCI. Establishing dynamic models to simulate the changes in gut microbiota at different time points and their effects on the pathological process of SCI will provide a more comprehensive understanding of their interaction patterns.

Building on these mechanistic insights, developing precise treatment plans is also an important direction for future research. Personalized gut microbiota modulation strategies should be developed based on the characteristics of gut microbiota in different individuals. Utilizing big data and artificial intelligence technologies for data analysis and predictive modeling, patient clinical information, gut microbiota data, and treatment responses should be integrated to establish diagnostic models that accurately predict patients’ responses to different treatment strategies, thus achieving personalized treatment. For example, by analyzing the composition and functions of patients’ gut microbiota, combined with the severity and type of their SCI, suitable probiotic strains, prebiotic formulations, or FMT donors can be precisely selected for patients to improve treatment outcomes. Strengthening the long-term monitoring of the safety and effectiveness of treatment strategies, establishing a comprehensive evaluation system, and tracking the long-term efficacy and adverse reactions of patients after treatment will provide reliable evidence for the optimization and promotion of these strategies.

Exploring combination therapy strategies is equally important. Research on the combined therapeutic effects and underlying mechanisms of gut microbiota modulation with existing SCI treatment methods, such as stem cell transplantation, gene therapy, and rehabilitation training, is essential (310, 311). The combination of gut microbiota modulation and stem cell transplantation may improve the gut microenvironment, enhance the survival and differentiation capacity of stem cells, and enhance neural repair (312, 313). The combination of gut microbiota modulation and rehabilitation training can further optimize rehabilitation outcomes by regulating the gut microbiota, improving patients’ nutritional absorption and immune functions, and providing a better physical foundation for rehabilitation training. Meanwhile, rehabilitation training can also promote the balance of gut microbiota, forming a positive feedback loop (314). Studying the synergistic mechanisms of combination therapy strategies provides theoretical support for developing more effective combined treatment plans.

## 7 Conclusion

In summary, there is a close and complex bidirectional relationship between the gut microbiota and SCI. SCI can lead to significant changes in the structure and function of the gut microbiota, which in turn can cause impaired intestinal barrier function, inflammatory responses, oxidative stress, neurological dysfunction, and a series of complications. The gut microbiota and its metabolites also significantly influence the repair of neurological function and both the occurrence and development of complications after SCI through metabolic and immune pathways. Treatments targeting the gut microbiota offer new strategies for managing SCI. FMT promotes the reconstruction of the gut microbiota and regulates the balance of the gut microbiota ecosystem; it has demonstrated positive effects in animal experiments, such as enhancing neurological recovery and improving intestinal barrier function. Probiotic interventions increase the abundance of beneficial bacteria, regulate the gut microbiota structure and intestinal barrier function, and support neurological recovery. Dietary interventions that adjust components—such as increasing the intake of high-fiber foods and prebiotics—can regulate the gut microbiota and improve gut function, thereby aiding the rehabilitation of SCI patients. In the future, research on the gut microbiota and SCI holds great potential. In-depth mechanistic studies, supported by multi-omics techniques, will help comprehensively elucidate the molecular mechanisms and signaling pathways underlying the interactions between the gut microbiota and SCI, providing a stronger theoretical foundation for treatment. Developing precise treatment plans and utilizing big data alongside artificial intelligence technologies to achieve personalized therapies will enhance treatment effectiveness. Moreover, exploring combined treatment strategies and investigating the synergistic effects of gut microbiota modulation with existing therapies hold promise for delivering more effective, comprehensive treatment options for SCI patients.
